# A possible pattern in the evolution of male meiotic cytokinesis in angiosperms

**DOI:** 10.1093/aobpla/plae017

**Published:** 2024-03-26

**Authors:** Mingli Hu, Zhanhong Ren, Ning Rong, Mei Bai, Hong Wu, Ming Yang

**Affiliations:** School of Pharmacy, Xianning Medical College, Hubei University of Science and Technology, Xianning 437100, China; Hubei Key Laboratory of Diabetes and Angiopathy, Medicine Research Institute, Xianning Medical College, Hubei University of Science and Technology, Xianning 437100, China; College of Life Sciences, South China Agricultural University, Guangzhou 510642, China; College of Life Sciences, South China Agricultural University, Guangzhou 510642, China; College of Life Sciences, South China Agricultural University, Guangzhou 510642, China; Department of Plant Biology, Ecology, and Evolution, Oklahoma State University, 301 Physical Sciences, Stillwater, Oklahoma 74078, USA

**Keywords:** Angiosperm evolution, climate cooling, Cretaceous, cytokinesis, male meiosis, microtubule cold sensitivity, phragmoplast

## Abstract

Evolution of cellular characteristics is a fundamental aspect of evolutionary biology, but knowledge about evolution at the cellular level is very limited. In particular, whether a certain intracellular characteristic evolved in angiosperms, and what significance of such evolution is to angiosperms, if it exists, are important and yet unanswered questions. We have found that bidirectional cytokinesis occurs or likely occurs in male meiosis in extant basal and near-basal angiosperm lineages, which differs from the unidirectional cytokinesis in male meiosis in monocots and eudicots. This pattern of cytokinesis in angiosperms seems to align with the distribution pattern of angiosperms with the lineages basal to monocots and eudicots living in tropical, subtropical or temperate environments and monocots and eudicots in an expanded range of environments including tropical, subtropical, temperate, subarctic and arctic environments. These two cytokinetic modes seem to result from two phragmoplast types, respectively. A phragmoplast in the bidirectional cytokinesis dynamically associates with the leading edge of a growing cell plate whereas a phragmoplast in the unidirectional cytokinesis is localized to an entire division plane. The large assembly of microtubules in the phragmoplast in unidirectional cytokinesis may be indicative of increased microtubule stability compared with that of the small microtubule assembly in the phragmoplast in bidirectional cytokinesis. Microtubules could conceivably increase their stability from evolutionary changes in tubulins and/or microtubule-associated proteins. Microtubules are very sensitive to low temperatures, which should be a reason for plants to be sensitive to low temperatures. If monocots and eudicots have more stable microtubules than other angiosperms, they will be expected to deal with low temperatures better than other angiosperms. Future investigations into the male meiotic cytokinetic directions, microtubule stability at low temperatures, and proteins affecting microtubule stability in more species may shed light on how plants evolved to inhabit cold environments.

## Teaser

Cytokinesis in plants is characterized by the formation of the cell plate. The cell plate results from the fusion of vesicles transported to the division site by the microtubular structure phragmoplast. In this paper, we provide evidence that male meiotic cytokinesis is bidirectional in angiosperms basal to monocots and eudicots and unidirectional in monocots and eudicots. Additional evidence from studies of microtubules in male meiosis strongly suggests that unidirectional cytokinesis results from a large phragmoplast persistently associating with the entire division plane and bidirectional cytokinesis from a small migrating phragmoplast associating only with the leading edge of the developing cell plate. Microtubules are very sensitive to low temperatures. We propose that the difference in cytokinetic direction or phragmoplast morphology reflects a difference in microtubule stability, which can potentially explain why basal and near-basal angiosperms live only in warm environments and monocots and eudicots live in both warm and cold environments.

## Bidirectional Cytokinesis As a Newly Discovered Feature in Early Angiosperm Species

Evolution of cellular structures is fundamental to the evolution of life forms. Studying cellular structures in taxa of evolutionary significance may reveal direct connections between molecular evolution and phenotypic evolution. Some differences in cellular structures clearly define large taxa. For example, the presence of the nucleus distinguishes eukaryotes from prokaryotes, and the presence of the plastid distinguishes photosynthetic eukaryotes  from non-photosynthetic eukaryotes. Another feature of a cellular structure in the evolution of seed plants is that pollen grains of ancient lineages have a single long germination aperture while later lineages have more germination apertures and/or a reduced aperture size. An interesting question is whether there are other cellular structures that exhibit an evolutionary pattern, and if such a pattern exists, how it may have impacted the evolution of the organisms in question.

**Figure 1.**
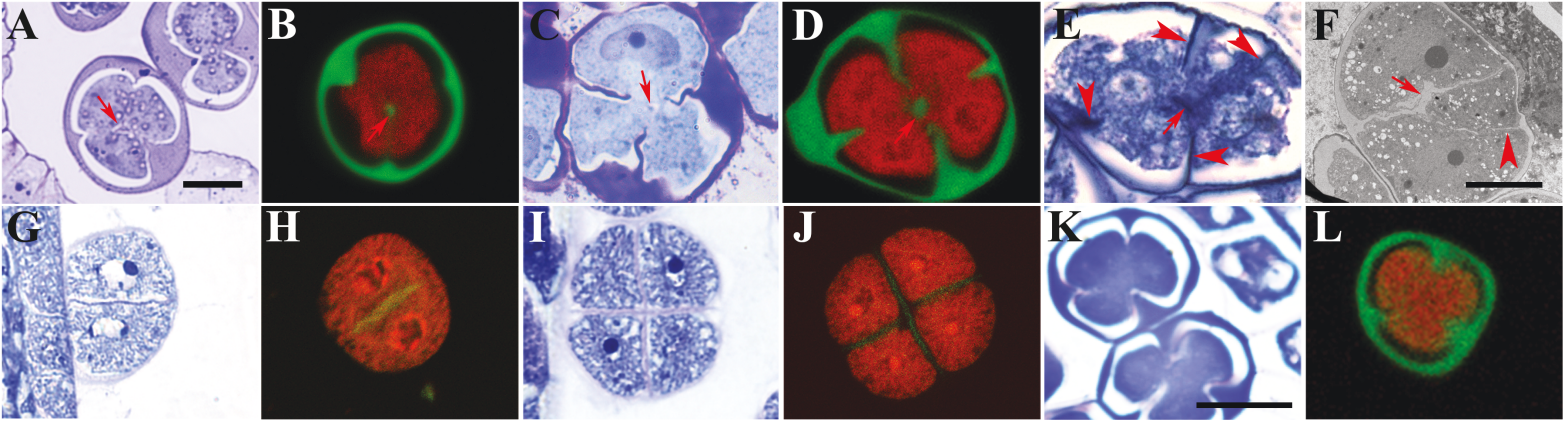
Male meiotic cytokinesis in five plant species. A, C, E, G, I and K, toluidine blue O-stained brightfield images. B, D, H, J and L, aniline blue and propidium iodide-stained confocal images. F, transmission electron microscopy image. A and B, *N. colorata*. C and D, *M. denudata*. E and F, *M. thorelii* Pierre. More than 50 microsporocytes at the cytokinesis stage were examined. A–F are examples of bidirectional cytokinesis. G–J, successive cytokinesis in *Oryza sativa* with G and H showing the first cytokinesis and I and J the second cytokinesis. It is an example of centrifugal cytokinesis. K and L, simultaneous cytokinesis in *Arabidopsis thaliana*. It is an example of centripetal cytokinesis. Arrows, cell wall islands. Arrowheads, centrifugal cell walls. A–E and G–J have the same magnification, and K and L have the same magnification. Details of the microscopy methods were previously described (Hu *et al.* 2021). Images of the cells shown in A, B and D were previously published (Hu *et al.* 2021; Wu *et al.* 2021).

**Figure 2.**
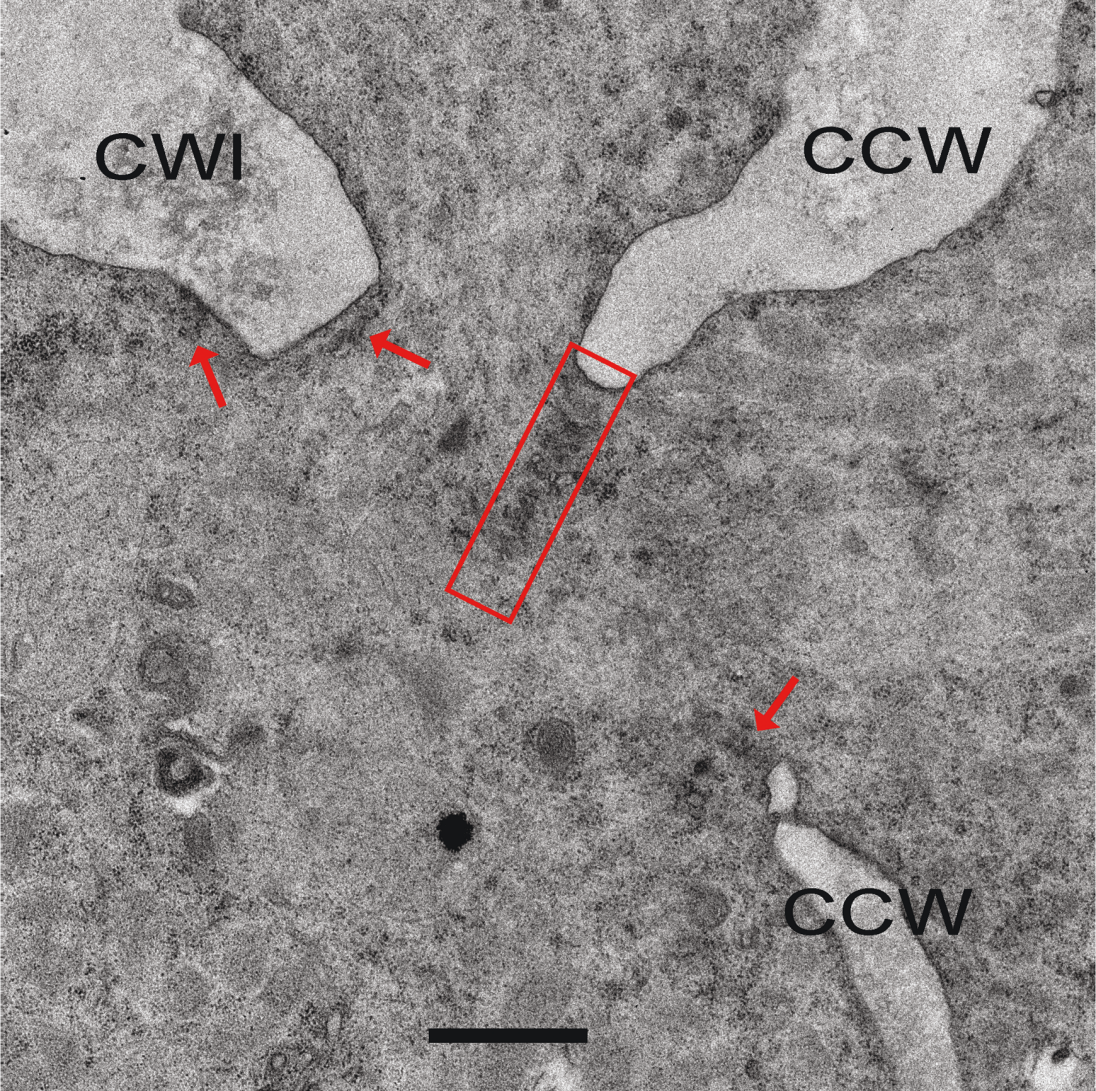
Transmission electron micrograph showing short cell plates in male meiotic cytokinesis in *Magnolia denudate*. The cell plates appeared to be in the form of a tubulo-vascular membrane network shown in Fig. 3. CCW, centripetal cell wall. CWI, cell wall island. Rectangle, relatively long cell plate. Arrows, short cell plates. Scale bar = 1 µm.

We recently reported that cytokinesis in male meiosis in basal angiosperm species *Nymphaea colorata* is bidirectional, that is, cytokinesis in microsporocytes occurs both centripetally from the cell periphery and centrifugally from a cell-wall island (CWI) in the centre of the cell (Wu *et al.* 2021; Fig. 1A and B). We also observed bidirectional cytokinesis in male meiosis in *Magnolia denudata* which belongs to Magnoliales, an ancient order of angiosperms (APG IV 2016; Hu *et al.* 2021; Fig. 1C and D). The microsporocytes in *N. colorata* and *M. denudata* clearly show the concurrent formation of cell walls from the cell periphery and the CWI (Fig. 1A–D; arrows, CWIs). The CWIs in these two species were bona fide cell walls because a space gap was created around them when plasmolysis of the cytoplasm occurred in the sample preparation process (Fig. 1A and C). The confocal images also show the discrete CWIs in the optical sections near the centres of these cells (Wu *et al.* 2021; Hu *et al.* 2021; Fig. 1B and D). We found a transmission electron microscopy image showing a similar CWI with concurrent and incomplete centripetal walls in male meiotic cytokinesis in *Nymphaea capensis* in an earlier publication (Figure 10 in Gabarayeva *et al.* 2001), suggesting that bidirectional cytokinesis is common in male meiosis in *Nymphaea* species. Moreover, using brightfield microscopy, transmission electron microscopy and fluorescence microscopy, we found that male meiotic cytokinesis in *Mitrephora thorelii* Pierre, a species in Annonaceae in Magnoliales, was also bidirectional with a CWI in the centre of the cell (Fig. 1E and F; arrowheads, incomplete centripetal cell walls; arrows, CWIs; Supporting Information—Fig. S1). In *M. denudata*, short cell plates were found to be associated with the leading edges of the centripetal cell walls and the edges of CWIs, but these cell plates were not connected to each other when the cytokinesis was in progress, making the CWI an island in the cytoplasm (Hu *et al.* 2021; Fig. 2). Bidirectional cytokinesis observed is independent of the cytokinesis types defined by the timing of cytokinesis, namely the simultaneous type in *N. colorata* (Wu *et al.* 2021) and *N. capensis* (Gabarayeva *et al.* 2001) and the intermediate type in *M. denudata* (Hu *et al.* 2021). These observations suggest that male meiotic bidirectional cytokinesis may be a common phenomenon in extant angiosperm lineages basal to monocots and eudicots that, in contrast, undergo unidirectional male meiotic cytokinesis (Fig. 1G–L). Moreover, we argue that this difference in cytokinetic direction may be indicative of an important difference in phragmoplast formation between these angiosperm groups, which may carry an important clue to the evolutionary process of angiosperms.

## Why Was Bidirectional Cytokinesis In Male Meiosis In Early Angiosperms Overlooked?

Male meiotic cytokinesis has been extensively studied in many angiosperm species that include some belonging to early angiosperm lineages, but bidirectional cytokinesis was not reported in the literature before our publications. In fact, a currently widely held view of male meiotic cytokinesis in angiosperms is that it is either centripetal or centrifugal (Furness *et al.* 2002; Nadot *et al.* 2008). We think that bidirectional cytokinesis was overlooked for the following reasons: (i) fewer studies on male meiotic cytokinesis in early angiosperm species were conducted; (ii) the CWI is a small and transient structure in the centre of the cell that can be missed due to insufficient sampling and/or sectioning and (iii) the microscopy methods were not suitable for detecting it. As to the third reason, it is particularly worth noting that fluorescent images of the callose walls cannot be reliably used to distinguish between unidirectional and bidirectional cytokinesis in male meiosis. Callose walls around the microspores may undergo uneven synthesis after cell plate formation, and they may be also subsequently subject to uneven degradation at the tetrad stage presumably due to varying degrees of exposure of different wall areas to the tapetum-derived callases in the locule (Ressayre *et al.* 2002; Wu and Yang 2005; Albert *et al.* 2011). A cell plate is essentially a structure of plasma membranes whose formation defines cytokinesis and precedes the deposit of wall materials outside the plasma membranes. The newly formed cell walls in male meiotic cytokinesis in plants, as represented by those in *Arabidopsis*, are callosic without cellulose, hemicellulose and pectin; only after cytokinesis at the tetrad stage hemicellulose, pectin and primexine materials are deposited onto the callose wall (Otegui and Staehelin 2004; Wang *et al.* 2021). A hydrophilic fluorescent dye that stains callose does not stain the cell plate, making identification of the cell plate difficult or impossible by fluorescence microscopy. However, under the bright field of a light microscope, a line or narrow region with little cell wall materials that partitions the cytoplasm during cytokinesis can be recognized as a cell plate. A cell plate can also be detected as a narrow electron-dense structure by transmission electron microscopy. Only by following how and when the cell plate is formed can the cytokinetic process be accurately characterized. We propose that brightfield microscopy or transmission electron microscopy should be used to determine whether the CWI exists during male meiotic cytokinesis. Our conclusions on the occurrences of bidirectional cytokinesis in *N. colorata*, *M. denudata* and *M. thorelii* were all based on brightfield microscopy and transmission electron microscopy images, with additional support from callose fluorescent images (Wu *et al.* 2021; Hu *et al.* 2021; Fig. 1E and F; Supporting Information—Fig. S1).

Even though the literature on male meiotic cytokinesis in angiosperms is not focussed on bidirectional cytokinesis, we still carefully examined the brightfield images of male meiocytes or tetrads available in publications. We found seven additional species in five angiosperm lineages basal to monocots and eudicots according to the current angiosperm phylogenetic tree (APG IV 2016), namely Annonaceae, Aristolochiaceae, Calycanthaceae, Magnoliaceae and Piperaceae, which potentially have bidirectional cytokinesis in male meiosis (Supporting Information—Tables S1 and S2). These past investigations were not focussed on catching the incomplete cell plate, but they seemed to show incomplete cell plates or walls and possibly a CWI in the microsporocytes. On the contrary, we did not find a case of incomplete cell plate or wall among 76 species belonging to 36 families of monocots and eudicots (Supporting Information—Table S1), whose cytokinesis is largely represented by that in rice (centrifugal; Fig. 1G–J) or *Arabidopsis* (centripetal; Fig. 1K and L). These observations further raise the possibility that bidirectional cytokinesis is an ancient form in male meiosis in angiosperms, and it evolved to be either centripetal or centrifugal in later lineages. It is noted here that Li *et al.* (2023) recently reported discontinuous cell walls in male meiotic cytokinesis in *Mitrephora tomentosa*, which we cited in Supporting Information—Table S2 as a species in Annonaceae likely with bidirectional cytokinesis. However, Li *et al.* (2023) mistook bidirectional callose deposition for bidirectional cytokinesis. Again, our definition of bidirectional cytokinesis is based on how the cell plate is formed. Publications just showing bidirectional callose deposition, or even mistaking uneven callose degradation for bidirectional callose deposition, do not address how the cell plate is formed.

## Do Differences in Microtubule Stability Underlie the Cytokinetic Differences?

**Figure 3.**
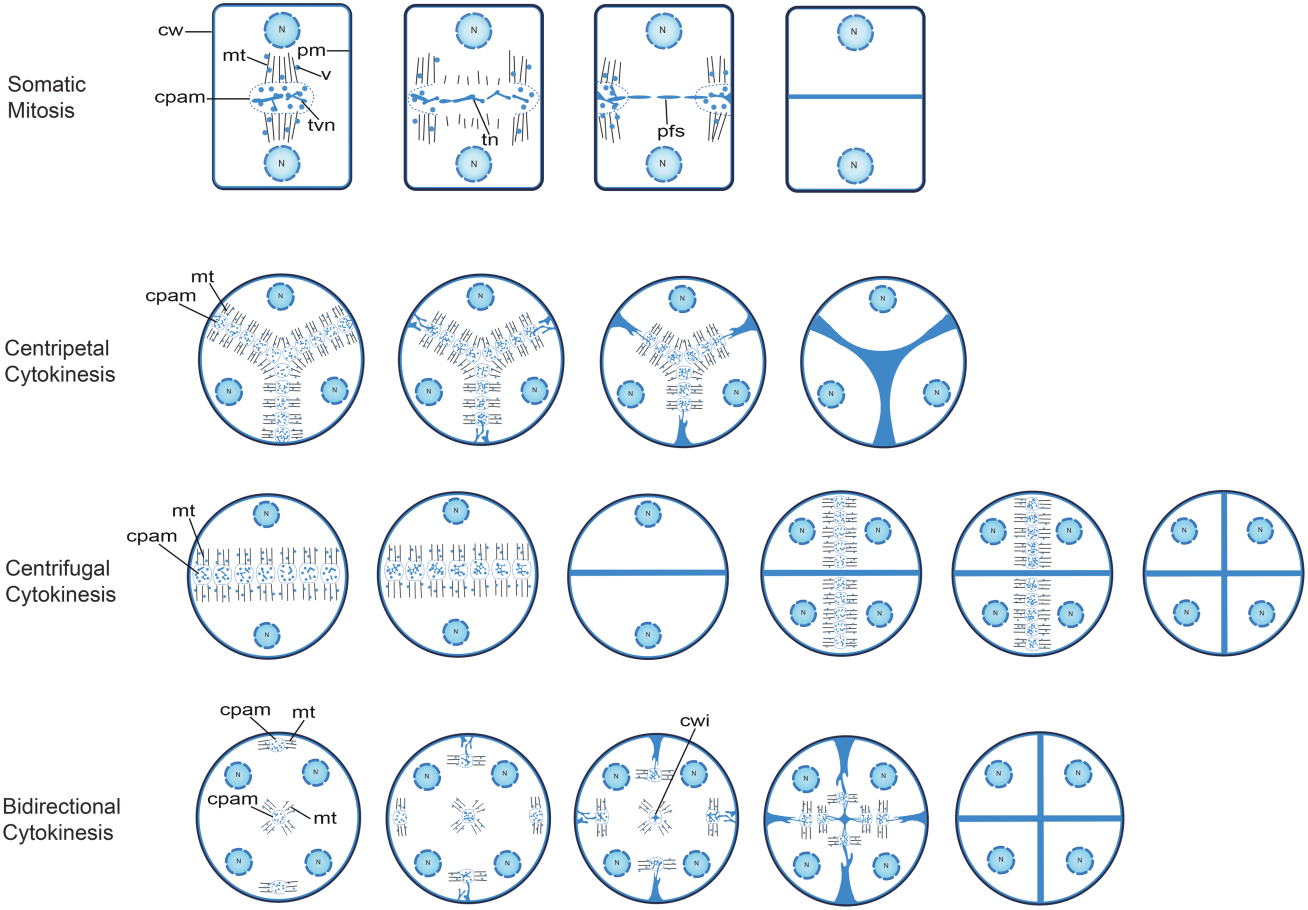
Schematic representation of cytokinetic patterns in somatic mitosis and male meiosis. In all cytokinetic patterns, the cell plate is formed from vesicles that are transported on microtubules of the phragmoplast. Male meiotic bidirectional cytokinesis of the simultaneous type, such as that in *N. colorata* (Wu *et al.* 2021) and *N. capensis* (Gabarayeva *et al.* 2001), was not drawn because no microtubular data are available, but it is expected to be similar to the bidirectional cytokinesis of the intermediate type shown here. The drawings were based on: somatic mitosis (Seguí-Simarro *et al.* 2004); centripetal cytokinesis (Otegui and Staehelin 2004); centrifugal cytokinesis (Sannier *et al.* 2006; Shamina *et al.* 2007; and Zhang *et al.* 2011); bidirectional cytokinesis (Brown and Lemmon 1992; Hu *et al.* 2021). cw, cell wall; pm, plasma membrane; N, nucleus; mt, microtubule; v, vesicle; cpam, cell plate assembly matrix; tvn, tubulo-vesicular membrane network; tn, tubular network; pfs planar fenestrated sheet and cwi, cell wall island.

Building a new cell wall in dividing plant somatic cells relies on the phragmoplast which is a microtubule-based module for constructing the initial cell wall, the cell plate (Smertenko 2018). In somatic mitosis, the microtubules from two opposite poles intercept at the division plane where the cell plate assembly matrix is located (Seguí-Simarro *et al.* 2004; Fig. 3). The vesicles in the cell plate assembly matrix fuse to first form tubulo-vesicular membrane networks, which turn into tubular networks upon further fusion between these membrane structures. The tubular networks develop into a planar fenestrated sheet, which is the mature form of the cell plate that eventually produces the new cell walls. During the centrifugal expansion of the developing cell plate in mitosis, microtubules in the centre of the phragmoplast disassemble and new microtubules assemble at the edge of the cell plate. In the centripetal (Otegui and Staehelin 2004; Fig. 3) and centrifugal types (Shamina *et al.* 2007; Zhang *et al.* 2011; Fig. 3) of meiotic cytokinesis, the basic features of cell plate formation from vesicles to the planar fenestrated sheet appear to be the same as in mitosis, except that a large or many mini-phragmoplasts occupy the entire division plane and their microtubules do not undergo the dynamic changes as in mitosis (De Storme and Geelen 2013). The early and late connections between the cell plate and the cell periphery give rise to the centripetal and centrifugal appearances, respectively (Otegui and Staehelin 2004; Sannier *et al.* 2006; Fig. 3). Meiotic bidirectional cytokinesis is similar to cytokinesis in mitosis in that the three phragmoplasts, two near the cell periphery and one in the cell centre, undergo dynamic changes to only associate with the growing edges of the developing cell plates (Brown and Lemmon 1992; Fig. 3).

Bidirectional cytokinesis in male meiosis involves concurrent phragmoplasts at the cell periphery and in the cell centre that expand toward each other to complete the cell plate (Brown and Lemmon 1992). The peripheral phragmoplast took the shape of a hollow cylinder, resembling the somatic phragmoplast. The central phragmoplast seemed to be a solid cylinder albeit a small one. In contrast, in male meiosis in monocots and eudicots, the shape of the phragmoplasts (also named a radial microtubule array or concurrent mini-phragmoplasts in simultaneous cytokinesis) is a large solid cylinder (Fig. 3; Otegui and Staehelin 2004; Sannier *et al.* 2006; Shamina *et al.* 2007; Zhang *et al.* 2011). It seems readily conceivable that the large solid cylinder-shaped phragmoplasts in male meiosis in monocots and eudicots could have evolved from increased stability of microtubules in the central and peripheral phragmoplasts in bidirectional cytokinesis, respectively.

## The Potential Significance of Increased Microtubule Stability in Angiosperm Evolution

Microtubules disassemble at low and yet above-freezing temperatures, making microtubules uniquely cold-sensitive when compared with the other cytoskeleton component, the microfilament (Li and Moore 2020). More stable microtubules can conceivably make a cell more tolerant to low temperatures than otherwise. Monocots and eudicots may have more stable microtubules than older angiosperm lineages, judging from the different male meiotic phragmoplast features in these groups of plants (Fig. 3). In particular, the phragmoplasts in monocots and eudicots should not undergo much microtubule disassembly so that they can attain a large solid cylinder shape. In the older angiosperm lineages, the peripheral phragmoplasts undergo concurrent microtubule disassembly and assembly in an outside-in manner to form a hollow cylinder and the small solid cylinder-like phragmoplast associated with the CWI never gets as large as the phragmoplasts in monocots and eudicots. De Storme and Geelen (2013) essentially arrived at the same conclusion when comparing the dynamics of male meiotic phragmoplasts in monocots and eudicots with that in mitosis since the phragmoplasts in *Magnolia* behave like those in mitosis (Brown and Lemmon 1992). Are microtubules in monocots and eudicots indeed more stable than in the other extant angiosperm species? Is there a correlation between the level of microtubule stability and the level of cold tolerance in angiosperms? The answers to these questions may be obtained by comparative studies of cold tolerance and microtubule stability at different temperatures in representatives of monocots, eudicots and other angiosperm species.

The evolutionary history of angiosperms coincided with the climate in the Cretaceous period (~145–66 mya). The Cretaceous period is known as one of the warmest periods in the history of Earth, but it still had temperature fluctuations. Its temperatures peaked around the middle of the period with two cold intervals before (~118–112 mya) (Herrle *et al.* 2015) and after (~83–66 mya) (Linnert *et al.* 2014) the temperature peak, respectively. If angiosperm evolution was tightly linked to the temperature fluctuations in Cretaceous, the global distributions of angiosperm lineages may exhibit a pattern on the angiosperm phylogenetic tree. To seek such a potential pattern, we surveyed the available information on the global distributions of all orders of angiosperms in regard to their naturally permissible growth temperatures. The results were then applied to the angiosperm phylogenetic tree (Supporting Information—Fig. S2; APG IV 2016). Our survey found that the seven oldest extant angiosperm orders basal to monocots and dicots in the phylogenetic tree, Amborellales, Nymphaeales, Austrobaileyales, Magnoliales, Laurales, Piperales and Canellales, live in tropical or tropical to warm temperate (here and hereafter tropical and other climatic terms refer only to the levels of temperature they typically represent) environments. On the other hand, early angiosperm pollen fossils were found only in low paleolatitude areas in the Early Cretaceous (145–100.5 mya, Crane and Lidgard 1989) and it was very warm around 125 mya when the earliest angiosperm macrofossil was found (Sun *et al.* 2002; Steuber *et al.* 2005; Coiffard *et al.* 2007). Living in warm environments, therefore, seems to be a common characteristic of the extant basal and near-basal angiosperms and extinct early angiosperms. The next orders, including Chloranthales and Ceratophyllales that appeared just before monocots and eudicots, respectively, are tropical to temperate, presumably emerged in response to a cold interval. Indeed, the first monocot order Acorales and the first eudicot order Ranunculales are tropical to temperate and tropical to subarctic, respectively, further supporting a cold interval as at least one of the driving forces for the origins of the immediate ancestors of monocots and eudicots, and the earliest monocots and eudicots themselves. The earliest fossil records for both monocots and eudicots are approximately 120 mya (Friis *et al.* 2006), which interestingly coincided with the cold interval of 118–112 mya in the Early Cretaceous (Herrle *et al.* 2015). This coincidence lends more support to the view that the early divergence of angiosperms may have been driven at least partially by an episode of global cooling.

Some of the later angiosperm lineages such as Arecales, Commelinales, Zingiberales, Pandanales, Picramniales, Huerteales, Dilleniales, Vahliales, Garryales, Metteniusales and Icacinales exhibit an ancestral trait in terms of the tropical to warm temperate distributions (Supporting Information—Fig. S2), which may be cases of reverse evolution due to the returning of a warm climate after the cooling interval in the Early Cretaceous. In addition, many latest orders in Supporting Information—Fig. S2 are tropical to subarctic or arctic and their representatives’ fossils were first detected in Late Cretaceous (Friis *et al.* 2006). By the same mechanism discussed so far, these youngest lineages, might have arisen from the aforementioned second and more extended cooling interval in the Late Cretaceous (Linnert *et al.* 2014). Therefore, the repeated global cooling episodes in Cretaceous might have been a determining factor in the evolution of angiosperms.

Plants are expected to rely on multiple mechanisms to cope with low temperatures. For example, in response to low temperatures, cold-tolerant plants undergo multiple changes at the molecular level to deal with the low temperatures, which include increasing unsaturated lipids in their cell membrane and hormonal, metabolic, transcriptional and proteomic changes involved in cold tolerance (Barrero-Sicilia *et al.* 2017; Ritonga and Chen 2020). The cold-sensitive property of microtubules as a limiting factor for growth, development and distribution of plants has been discussed before (Nick 2000). As reviewed by De Storme and Geelen (2013), meiotic cell plate formation, in both the centripetal and centrifugal cytokinesis, is extremely sensitive to cold stress, which can lead to defective phragmoplasts and hence a cytokinetic failure. Although it has not been investigated, meiotic phragmoplasts in bidirectional cytokinesis may be even more sensitive to low temperatures than those in centripetal and centrifugal cytokinesis, given the perceived more dynamic phragmoplasts in bidirectional cytokinesis than in centrifugal and centripetal cytokinesis. Microtubules are dynamic structures, that is, they undergo continual assembly and disassembly. Microtubule-associated proteins in animals and plants have been found to confer cold stability of microtubules by promoting assembly (tubulin polymerization) and/or suppressing disassembly (tubulin depolymerization) of microtubules (Cyr and Palevitz 1989; Rutten *et al.* 1997; Nick 2000; Wicker-Planquart *et al.* 2004; Mao *et al.* 2005; Li *et al.* 2009; Meng *et al.* 2010; Goodson and Jonasson 2018). It has been shown that enhanced expression of MICROTUBULE-ASSOCIATED PROTEIN 65-1 in soybean reduced cytosol ion leakage under freezing conditions, suggesting that this protein can be a positive factor for soybean cold tolerance (Kim *et al.* 2024). More stable microtubules could also result from evolutionary changes in the tubulin proteins that are building blocks of microtubules (Nick 2000). Could more stable microtubules be one of the key weapons that monocots and eudicots had for dealing with cold living conditions and the earlier angiosperm lineages did not have? If the answer is yes, considering the importance of microtubules for many cell division and morphogenic processes, more stable microtubules should impact the plant life far beyond cytokinesis. However, studying cytokinesis in male meiosis does provide a window for looking into the possible connections between the directions of cytokinesis and phragmoplast morphology and dynamics, and between microtubule stability and angiosperm evolution. Further comparative studies of cytokinesis, microtubule stability in phragmoplasts and other cytoskeletal structures under different temperatures, and relevant genes between warm- and cold-adapted orders and between warm- and cold-adapted taxa within the same order may shed light on how microtubules and their associated angiosperm lineages have evolved and what they may become in the future.

## Supporting Information

The following additional information is available in the online version of this article –

Table S1. Cytokinetic directions in various families of angiosperms.

Table S2. Additional species possibly undergo bidirectional cytokinesis in male meiosis.

Figure S1. More examples of microsporocytes in *M. thorelii* at early cytokinesis.

Figure S2. Natural temperature ranges for growth of angiosperms on a phylogenetic tree.

plae017_suppl_Supplementary_Figures

plae017_suppl_Supplementary_Table_S1

plae017_suppl_Supplementary_Table_S2

## Data Availability

Data sharing is not applicable to this article as all data are already contained within this article or in the Supporting Information.
