# Supplementary material for: A possible pattern in the evolution of male meiotic cytokinesis in angiosperms
Source: AoB Plants. 2024 Mar 26;16(2):plae017. doi: 10.1093/aobpla/plae017 (PMC10998459; doi:10.1093/aobpla/plae017)
Supplement: plae017_suppl_Supplementary_Figures [file plae017_suppl_supplementary_figures.docx]

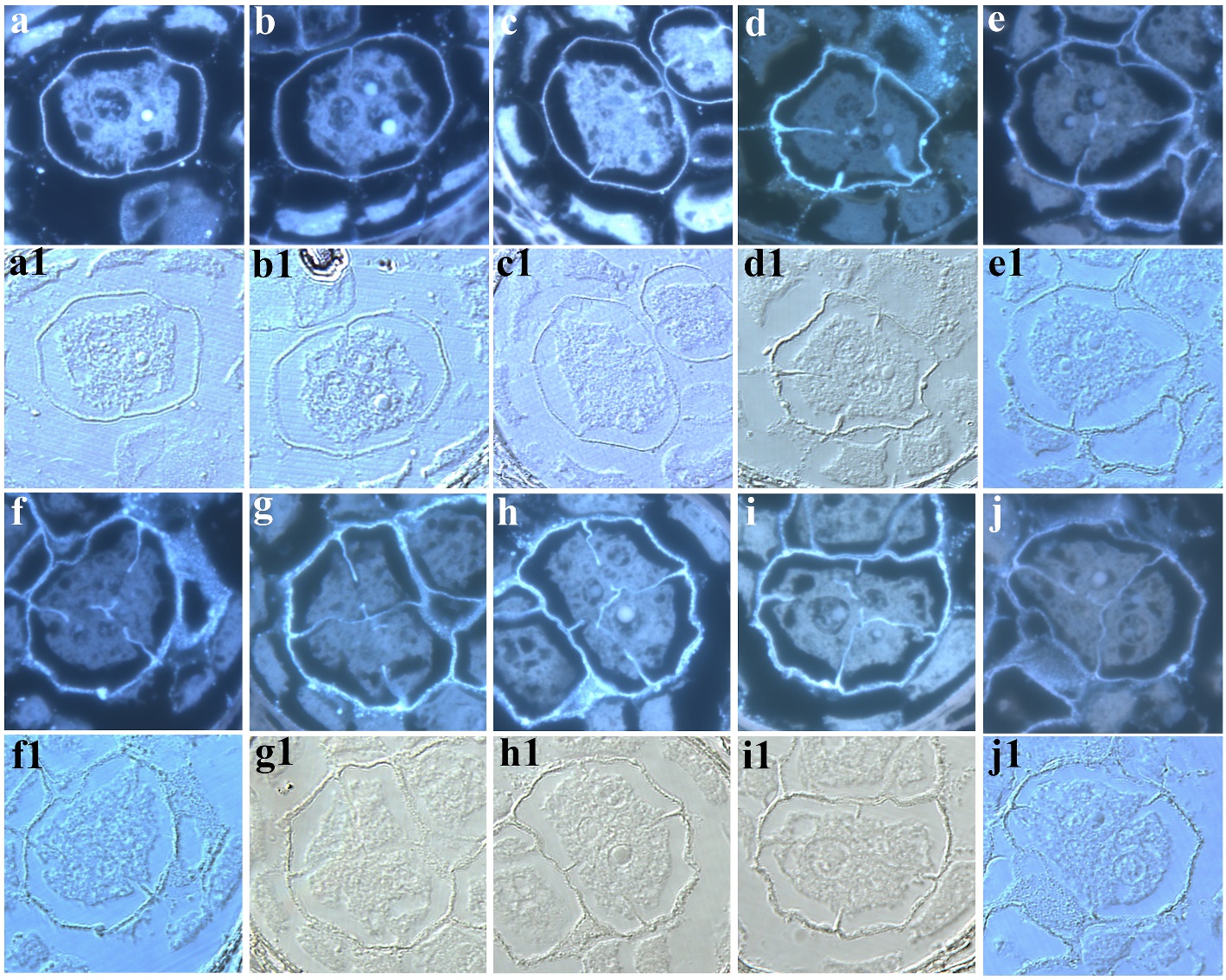


**Figure S1.** More examples of microsporocytes in *Mitrephora thorelii* at early cytokinesis. a-j, fluorescent images of microsporocytes in thin sections (1μm thick) stained with aniline blue (0.005%). a1-j1, differential interference contrast (DIC) microscopy images of a-f, respectively. The newly formed callose walls are thin and incomplete. A cell wall island (CWI) can be seen in d-f (arrows). In other cells, the CWI had connected with one or more centripetal walls. The large spatial gaps between the tapering ends of the thin centripetal walls and the CWIs in the fluorescent images and the absence of complete cytoplasmic partitioning in the DIC images indicate that the cell plates were progressing in a bidirectional manner. Scale bar in j1 = 10 µm for all images.


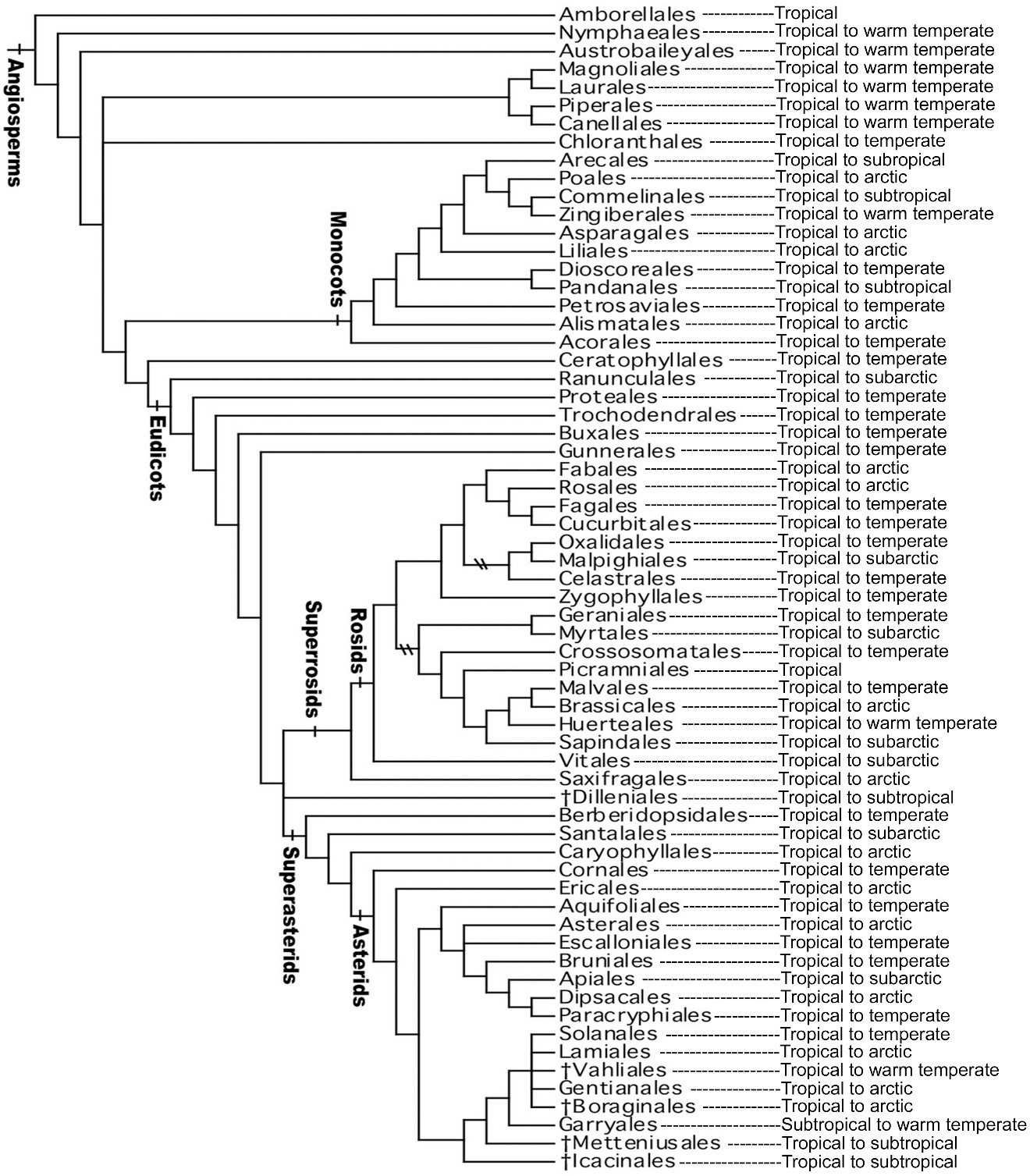


**Figure S2.** Natural temperature ranges for growth of angiosperms on a phylogenetic tree. The figure was adapted from (APG IV, 2016). The temperatures are defined by what the climatic terms typically refer to.
