# Supplementary material for: A possible pattern in the evolution of male meiotic cytokinesis in angiosperms
Source: AoB Plants. 2024 Mar 26;16(2):plae017. doi: 10.1093/aobpla/plae017 (PMC10998459; doi:10.1093/aobpla/plae017)
Supplement: plae017_suppl_Supplementary_Table_S2 [file plae017_suppl_supplementary_table_s2.docx]

| **Species** | **Reference** | **Evidence in reference** |
| --- | --- | --- |
| *Pseuduvaria trimera*  *Mitrephora tomentosa*  *Aristolochia steuensii*  *Asarum eumpaeum*  *Calycanthus floridus*  *Magnolia tripetala*  *Piper ornatum* | Li and Xu, Protoplasma, 2019, 256:53-68  Li et al., Diversity, 2023, 15, 898  González et al., 2001, Bot J Linn Soc, 137: 221-242  González et al., 2001, Bot J Linn Soc, 137: 221-242  Albert et al., Am J Bot, 2011, 98:189-96  Brown and Lemmon, 1992, J Cell Sci, 103, 1031–1038  Nadot et al., Am J Bot, 2008, 95:1426-36 | Figure 3  Figure 4g and i  Figure 35  Figure 21  Figure 21  Figure 18  Figure 2E |

**Table S2. Additional species possibly undergo bidirectional cytokinesis in male meiosis**
